# Mouse primary microglia respond differently to LPS and poly(I:C) in vitro

**DOI:** 10.1038/s41598-021-89777-1

**Published:** 2021-05-17

**Authors:** Yingbo He, Natalie Taylor, Xiang Yao, Anindya Bhattacharya

**Affiliations:** 1 Janssen Research & Development, LLC., Neuroimmunology Drug Discovery, 3210 Merryfield Row, San Diego, CA 92121 USA; 2 Janssen Research & Development, LLC., NonClinical Safety, San Diego, CA USA

**Keywords:** Cell biology, Neuroscience

## Abstract

Microglia, CNS resident innate immune cells, respond strongly to activation of TLR3 and TLR4, which recognize viral dsRNA poly(I:C) and bacterial endotoxin LPS, respectively. However, few studies have thoroughly and parallelly compared functional phenotypes and downstream mechanisms between LPS- and poly(I:C)-exposed primary microglia. Here, we investigated the responses of mouse primary microglia upon LPS and poly(I:C) stimulation by detecting various phenotypes ranging from morphology, proliferation, secretion, chemotaxis, to phagocytosis. Furthermore, we explored their sequential gene expression and the downstream signal cascades. Interestingly, we found that the microglial activation pattern induced by LPS was distinguished from that induced by poly(I:C). Regarding microglial morphology, LPS caused an ameboid-like shape while poly(I:C) induced a bushy shape. Microglial proliferation was also facilitated by LPS but not by poly(I:C). In addition, LPS and poly(I:C) modulated microglial chemotaxis and phagocytosis differently. Furthermore, genome-wide analysis provided gene-level support to these functional differences, which may be associated with NF-κb and type I interferon pathways. Last, LPS- and poly(I:C)-activated microglia mediated neurotoxicity in a co-culture system. This study extends our understanding of TLR roles in microglia and provides insights into selecting proper inflammatory microglial models, which may facilitate identification of new targets for therapeutic application.

## Introduction

Microglia, the resident macrophages in the brain, play critical roles in brain innate immunity, homeostasis, as well as in several neuroinflammatory pathologies^1–3^. Under physiological conditions, microglia maintain brain homeostasis by constantly surveying their surrounding micro-environment with highly motile processes^4^. Once being exposed to danger signals such as neurodegenerative debris, proinflammatory cytokines, injury, and cellular stress, microglia become activated, retract their processes, proliferate, and transform from ramified morphology to ameboid shape^5^. In addition, they can alter their gene expression patterns and release functional molecules such as pro- and anti-inflammatory cytokines, reactive oxygen species, and nitric oxide^6,7^. To exert their protective roles, microglia migrate towards the injury site directed by chemotactic signals, as well as phagocytose pathogens, microbes, toxic molecules, and cell debris^8,9^. With all these functions, microglia become the first line of defense in protecting central nervous system against harmful insults by modulating local immune responses.

Microglial functions under various circumstances may reflect their diverse phenotypes that acquire different activation signals^10^. Toll-like receptors (TLRs) are a conserved diverse receptor family that drives innate immune responses following the interaction with pathogen-associated molecular patterns (PAMPs), such as bacteria, fungi, and viruses^11,12^. To date, 10 functional TLRs have been identified in human and 12 in mouse^13^. Each TLR recognizes a specific type of PAMPs^14^. For example, TLR3 recognizes double-stranded RNA that is generated during the replication of RNA virus^15^; whereas TLR4 is the major receptor for LPS, a main component of the gram-negative bacterial cell wall^16^. Studies have demonstrated key roles of TLRs in neurological diseases^17^. Deficiency of TLR4 in microglia resulted in reduced microglial activation and increased Aβ deposition^18^; on the other hand, stimulation of TLR4 by LPS activated microglia and reduced p-Tau in the cortex^19^. Moreover, patients with major depressive disorder showed an elevated expression level of TLR3, which could be significantly counteracted by antidepressant treatments^20^. Therefore, TLRs probably represent potential pharmacological targets for the development of neuroprotective drugs, and investigation of the mechanism of TLR signaling may be essential for deciphering the roles of microglia in pathogenesis of neurological and neuropsychiatric diseases.

Microglia express all known TLRs and respond robustly to LPS and poly(I:C), a synthetic analogue of double-stranded RNA^21^. Individual studies have intensively reported the responses of microglia to either LPS or poly(I:C)^22–26^. However, the data directly and comprehensively comparing the effects of LPS *versus* poly(I:C) on phenotypes and function of microglia are limited. Reimer, et al. found that in human macrophages, LPS and poly(I:C) induced distinct cytokine responses through NF-κB and IRF3 signaling, respectively^27^. Different gene expression patterns and cytokine responses to LPS and poly(I:C) were also observed in immortal murine microglial BV2 cells^6,28^, although limitations of this cell line as microglia were demonstrated in our previous study^29^. Yet to date, no study has thoroughly compared and validated transcriptional profiles, signaling cascades, and functional phenotypes of cultured primary microglia in response to LPS and poly(I:C). A comprehensive understanding of these response differences is critical for determining an appropriate in vitro inflammation model for microglial biology.

To this end, we investigated and compared the immune responses of primary microglia to LPS and poly(I:C) by assessing multiple functional phenotypes ranging from morphology, proliferation, secretion, chemotaxis, to phagocytosis. The downstream mechanisms including consequential gene expression and signaling cascades were also explored. To test the interaction of microglia with neurons and mimic in vivo neuroinflammation, we developed a co-culture system. As a result, we found that LPS and poly(I:C) induced distinct microglial activation patterns, which is consistent with functional prediction based on differentially expressed gene profiles. Furthermore, this distinction may also attribute to NF-κb pathway and type 1 interferon pathway. Last, LPS- and poly(I:C)-activated microglia caused neurotoxicity in the co-culture system. Hence, this study extends our understanding of TLR roles in primary microglia and facilitates selection of appropriate in vitro models for microglia-based drug discovery.

## Results

### Both LPS and poly(I:C) change microglial morphology but only LPS enhances microglial proliferation

**Figure 1:**
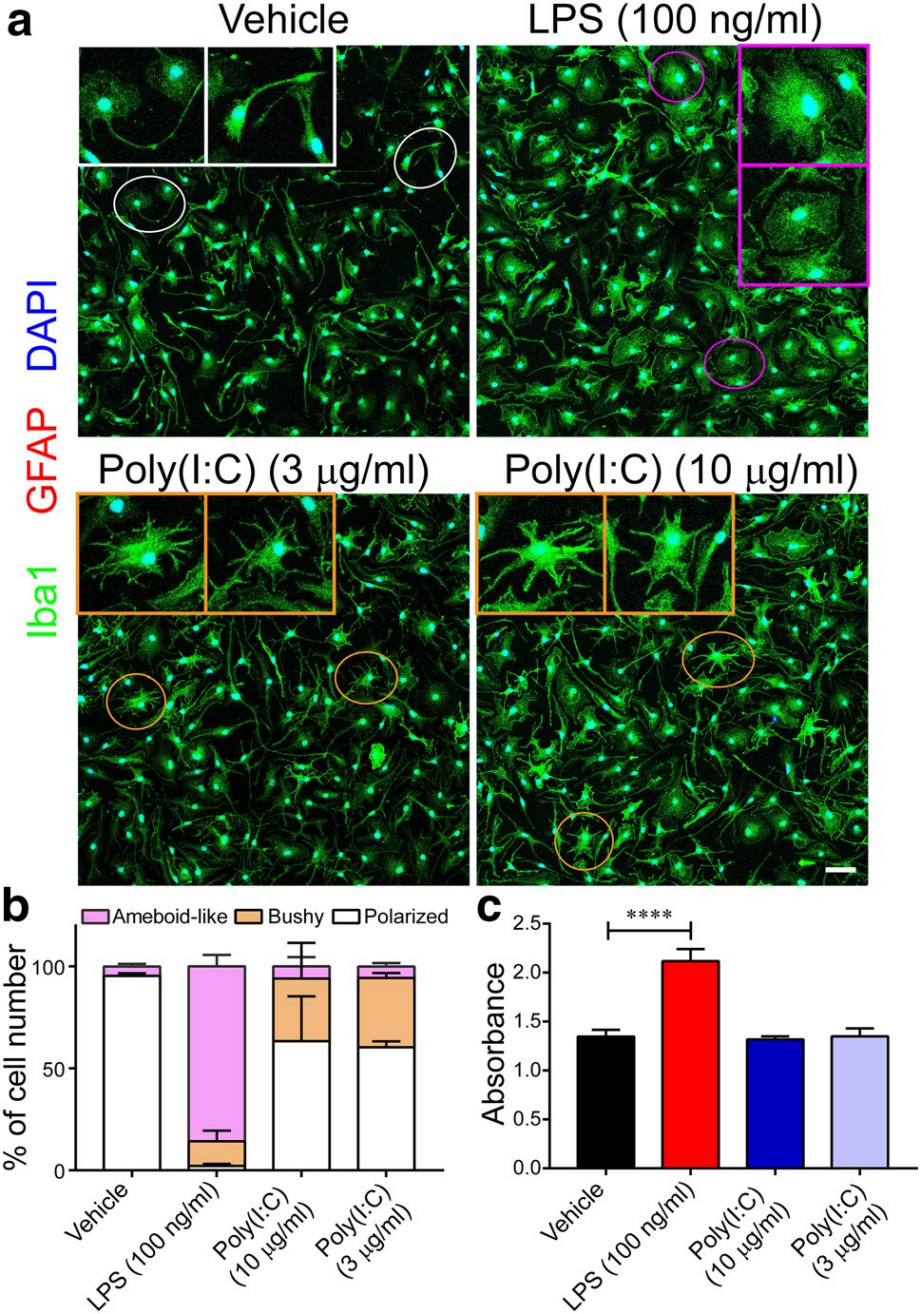
Morphological changes and proliferation of microglia in response to LPS or poly(I:C). Microglia were treated with 100 ng/ml LPS, 10 µg/ml poly(I:C), or 3 µg/ml poly(I:C) for 24 h. (**a**) Representative images of double immunofluorescence labeling for GFAP (red) and Iba1 (green) under the indicated conditions. Nuclei were counterstained with DAPI (blue). Different shapes of microglia were highlighted in circles: polarized (white); ameboid-like (purple); and bushy (yellow). Inserted are magnified images of the circle areas. Scale bar, 50 µm. (**b**) Ameboid-like, bushy, or polarized Iba1^+^ microglia were quantified with at least 3 randomly selected fields for each condition. Data are shown as means + SD. (**c**) The number of microglia after treatments was quantified by using CCK-8 kit. Data are shown as means + SD. Experiments were performed in six replicates and repeated three times independently. One-way ANOVA followed by the Tukey's post hoc test. ****P < 0.0001.

Pathological insults activate microglia and change their morphology both in vitro and in vivo^30^. To assess morphological changes induced by TLR ligands, we treated microglia with 100 ng/ml LPS, or 3 or 10 µg/ml poly(I:C). Untreated Iba1^+^ microglia exhibited polarized shape, indicating a baseline state (Fig. 1a). In contrast, LPS-treated microglia retracted their processes and changed morphology to ameboid-like shape with an increased cell number (Fig. 1a). On the other hand, microglia treated with poly(I:C) displayed a mixture of cell morphologies including bushy shape with more branches of processes compared to non-treated microglia, possibly indicating an alternative activation state (Fig. 1a). After quantification of these microglial morphologies under different conditions, we found that more than 90% of non-treated microglia showed polarized morphology, and more than 80% of LPS-treated microglia had ameboid-like morphology. However, in the cells treated with poly(I:C), less than 10% of the microglia were ameboid like-shaped, more than 50% maintained polarized morphology, and approximately 30% exhibited bushy morphology (Fig. 1b). These data suggest that microglia are more sensitive to LPS than poly(I:C) in inducing an ameboid-like morphology. In addition to morphological assessment, we measured cell number under each condition. Consistent with the results of immunostaining, treatment with LPS caused a 50% increase in cell number, whereas poly(I:C) showed no effects (Fig. 1c). Thus, the effects of LPS and poly(I:C) on microglial morphology and proliferation are different, suggesting that they activate microglia through different pathways.

### LPS and poly(I:C) regulate microglial transcription

**Figure 2:**
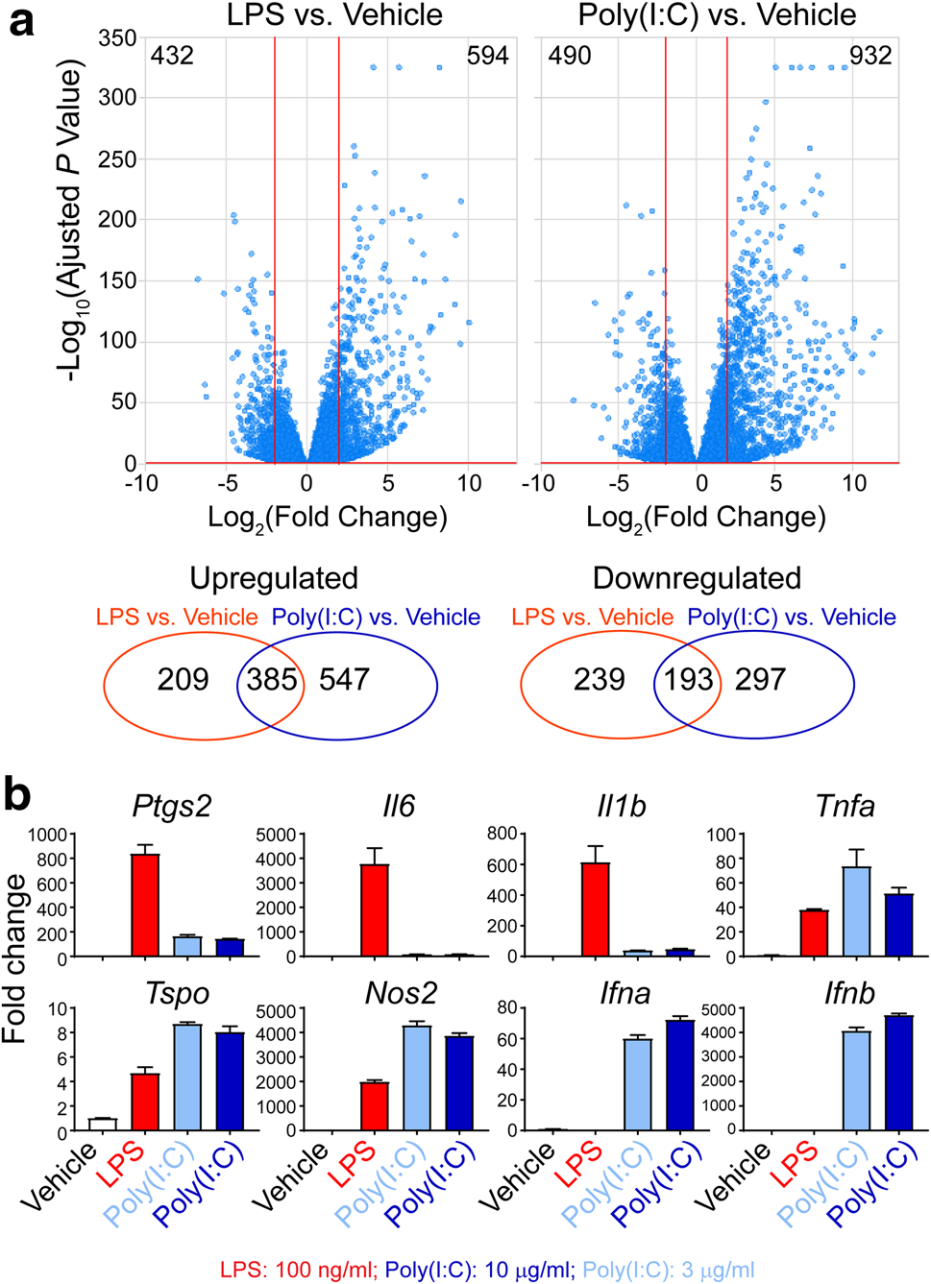
RNA-sequence analysis showing transcriptional changes in microglia induced by LPS or poly(I:C). Microglia were treated with 100 ng/ml LPS, 10 µg/ml poly(I:C), or 3 µg/ml poly(I:C) for 24 h followed by RNA extraction. (**a**) Volcano plots of genes present the magnitude (log_2_ (fold change), x-axis) and significance (− log_10_ (adjusted *P* value), y-axis) for LPS- or poly(I:C)-treated microglia, as compared to non-treated microglia. Each spot represents a transcript. The horizontal red line represents statistical significance threshold (adjusted *P* value < 0.05). Two vertical red lines represent the threshold of fold changes (log_2_ (fold change) > 2 or <  − 2). The number of significantly different transcripts was indicated in the corner. Venn diagram shows similarities and differences of up- and down-regulated transcripts between LPS- and poly(I:C)-treated microglia compared to non-treated microglia. (**b**) Selected gene expression in microglia following LPS or poly(I:C) treatment was verified by qRT-PCR. Data were normalized to *Gapdh* mRNA and expressed relative to vehicles.

Since microglia showed separate morphological and proliferative responses to LPS and poly(I:C), we sought to investigate global transcriptional expression in response to stimulation of these two TLR ligands. Following the treatments of LPS and poly(I:C), microglia were extracted for RNA-sequencing. For each sample, we obtained more than 90 million reads, in which more than 85% were uniquely mapped and paired (Supplementary Fig. S1), indicating high quality of sequencing data. Pairwise correlation analysis of the whole transcriptome profiles across all samples showed high correlation thus high consistency within groups and low correlation across groups (Supplementary Fig. S2). Furthermore, we found that in LPS-treated microglia, 594 genes showed at least fourfold higher expression and 432 genes at least fourfold lower expression compared to those in non-treated cells (Fig. 2a). In addition, in poly(I:C)-treated microglia, 932 genes exhibited at least fourfold increase and 490 genes at least fourfold decrease compared to those in non-treated cells (Fig. 2a). Among the changed genes, 385 upregulated and 193 downregulated genes were in common between LPS- and poly(I:C)-treated microglia, while the others were induced uniquely by LPS or poly(I:C) (Fig. 2a). The top common and uniquely regulated genes in LPS- and poly(I:C)-exposed microglia were also listed (Supplementary Table S1). These findings reflect similarity and differences in microglial gene expression profile upon the two distinct stimulation. To validate RNA-sequencing results, we selected and measured a panel of inflammation-related genes in microglia with or without LPS and poly(I:C) treatments by quantitative real-time PCR (qRT-PCR). Indeed, some of them, such as *Tnfa*, *Tspo*, and *Nos2*, were strongly upregulated upon both LPS and poly(I:C) treatments; whereas some other genes were predominantly induced by LPS (e.g. *Ptgs2*, *Il6*, and *Il1b*) or only by poly(I:C) (e.g. *Ifna* and *Ifnb*) (Fig. 2b). Therefore, microglia exhibited distinct gene profiles when exposed to LPS and poly(I:C).

### Functional analysis of differentially expressed genes between LPS- and Poly(I:C)-treated microglia

**Figure 3:**
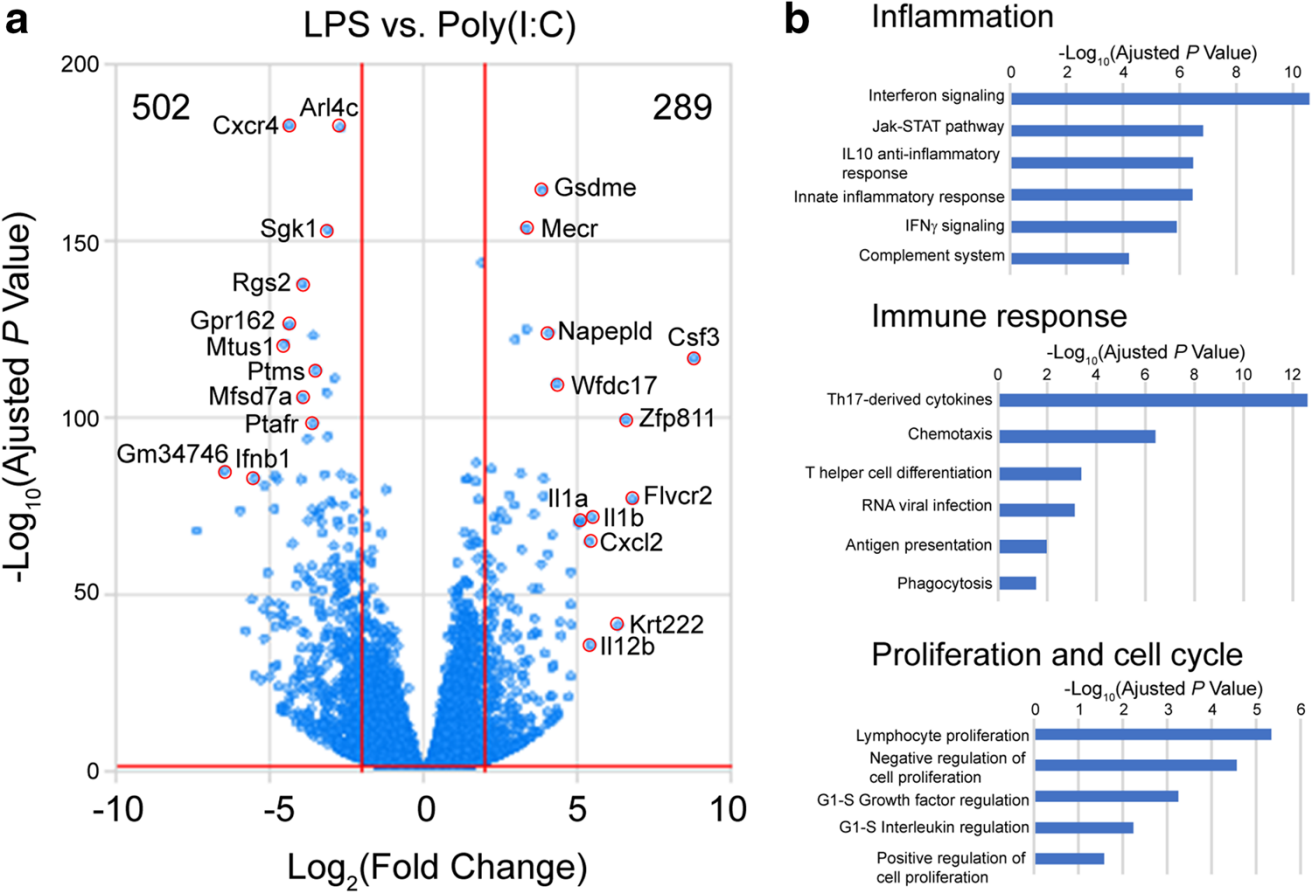
Transcriptional comparison between LPS- and poly(I:C)-treated microglia. (**a**) Volcano plots of genes present the magnitude (log_2_ (fold change), x-axis) and significance (− log_10_ (adjusted *P* value), y-axis) for LPS-treated microglia compared to those treated with poly(I:C). Each spot represents a transcript. The horizontal red line represents statistical significance threshold (adjusted *P* value < 0.05). Two vertical red lines represent the threshold of fold changes (log_2_ (fold change) > 2 or <  − 2). The number of significantly different transcripts was indicated in the corner. Top regulated transcripts are labeled with names. (**b**) Biological processes and pathways including inflammation, immune response, and proliferation and cell cycle enriched in LPS-treated microglia.

To further predict differences in biological functions and canonical pathways of microglia in response to LPS and poly(I:C), we compared differentially expressed genes under these two treatment conditions. A total of 791 genes with significantly differential expression were identified, including 289 upregulated genes with at least fourfold higher expression and 502 downregulated genes with at least fourfold lower expression in LPS-treated microglia *versus* those treated with poly(I:C) (Fig. 3a). By GeneOntology analysis, these differentially expressed genes were enriched in several categories such as inflammation, immune response, and proliferation and cell cycle (Fig. 3b), which are consistent with our previous observation that only LPS enhanced microglial proliferation (Fig. 1). In more detail, the interferon signaling and JAK-STAT pathway in inflammation category were significantly distinguishable between LPS- and poly(I:C)-treated microglia (Fig. 3b). In immune response category, chemotaxis and phagocytosis, the two most critical functions of microglia, were also differently affected by LPS and poly(I:C) (Fig. 3b). Together, these results provide transcriptional evidence elaborating functional and signaling differences of microglia in response to LPS and poly(I:C).

### Microglial secretion patterns in response to LPS or poly(I:C)

**Figure 4:**
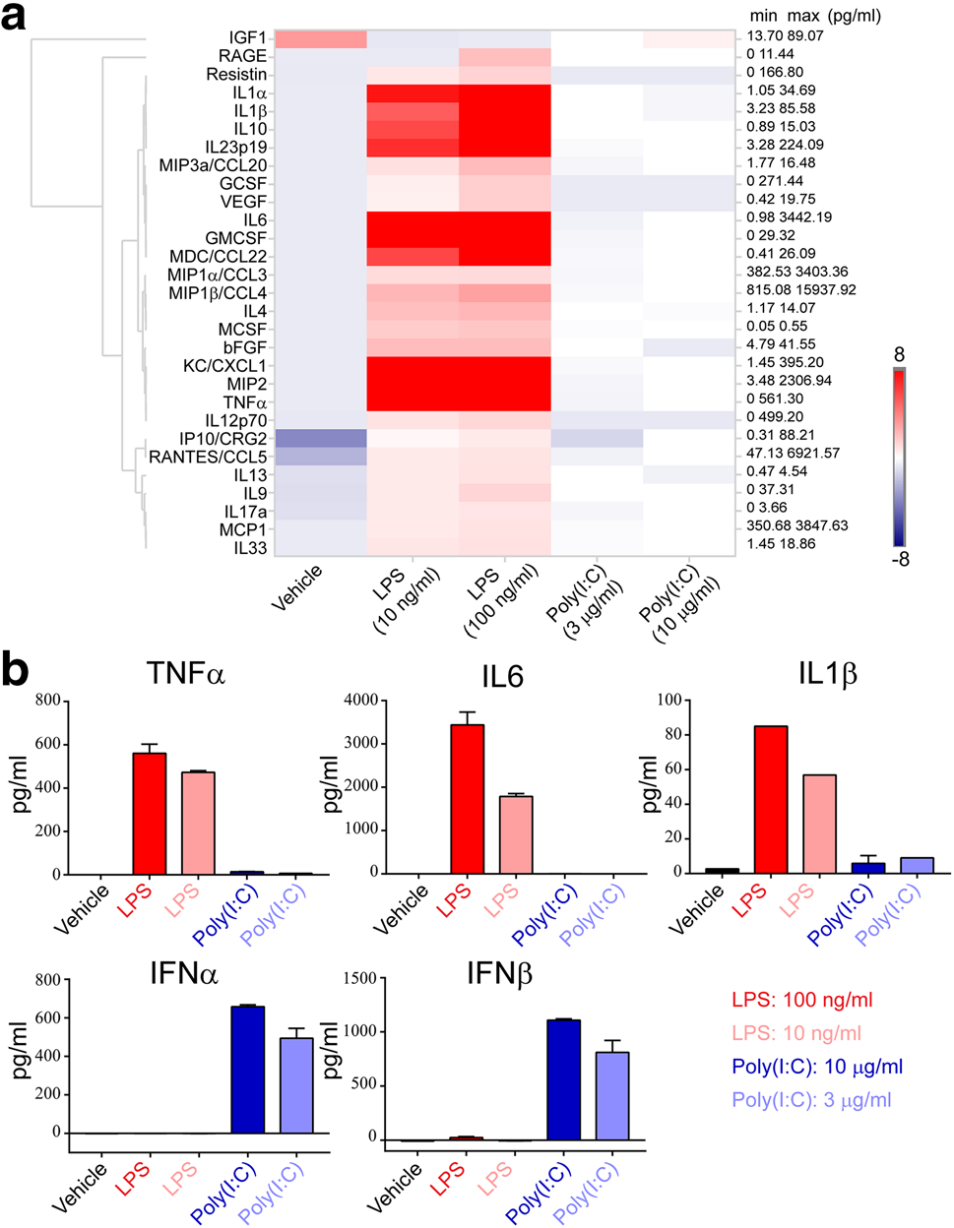
Protein secretion of microglia in response to LPS or poly(I:C). Microglia were treated with 100 ng/ml LPS, 10 ng/ml LPS, 10 µg/ml poly(I:C), or 3 µg/ml poly(I:C) for 24 h followed by media collection. (**a**) Heat map and hierarchical clustering of detectable 29 molecules that were released from the treated microglia and measured by Luminex multiplex. The minimal and maximal secretion level of each molecule was indicated. The heat map was created by QIAGEN OmicSoft Suite (version 10.0, https://digitalinsights.qiagen.com/products-overview/discovery-insights-portfolio/qiagen-omicsoft/). (**b**) The release of selected proteins from the treated microglia was verified by ELISA. Data are shown as means + SD. Experiments were repeated twice independently.

Cytokines and chemokines, mainly produced by microglia in brain, are critically involved in neuroinflammatory processes^31^. Next, we examined secretion of cytokines and chemokines in microglia in response to LPS or poly(I:C) by using a customized multiplex Luminex assay. Of 29 detectable inflammation-related molecules in the selected panel, the majority showed a significantly higher secretion level upon stimulation by 10 or 100 ng/ml LPS compared to vehicle control (Fig. 4a). In contrast, much less secretion of these molecules was detected in response to 3 or 10 µg/ml poly(I:C) treatment, although their secretion levels were slightly higher than those in non-treated cells (Fig. 4a). For further confirmation, we selected and measured the levels of three pivotal cytokines including TNFα, IL6, and IL1β by ELISA. In line with the results of multiplex Luminex assay, the release of these three cytokines were significantly enhanced by LPS concentration-dependently (Fig. 4b). However, low or no release of these molecules was found in poly(I:C)-treated microglia (Fig. 4b). According to the pathway analysis that interferon signaling is one of the most differential pathways between LPS and poly(I:C) treatments, we thus compared IFNα and IFNβ secretion in microglia as well. Unlike most molecules with a higher secretion level upon LPS stimulation, the secretion of both molecules was only strongly induced by poly(I:C) in a concentration-dependent manner (Fig. 4b). The results were not only consistent with our gene expression data but also supported the gene-based pathway analysis. Therefore, microglia selectively respond to LPS and poly(I:C) by secreting distinct molecules at different levels.

### Microglial chemotaxis in response to LPS and poly(I:C)

**Figure 5:**
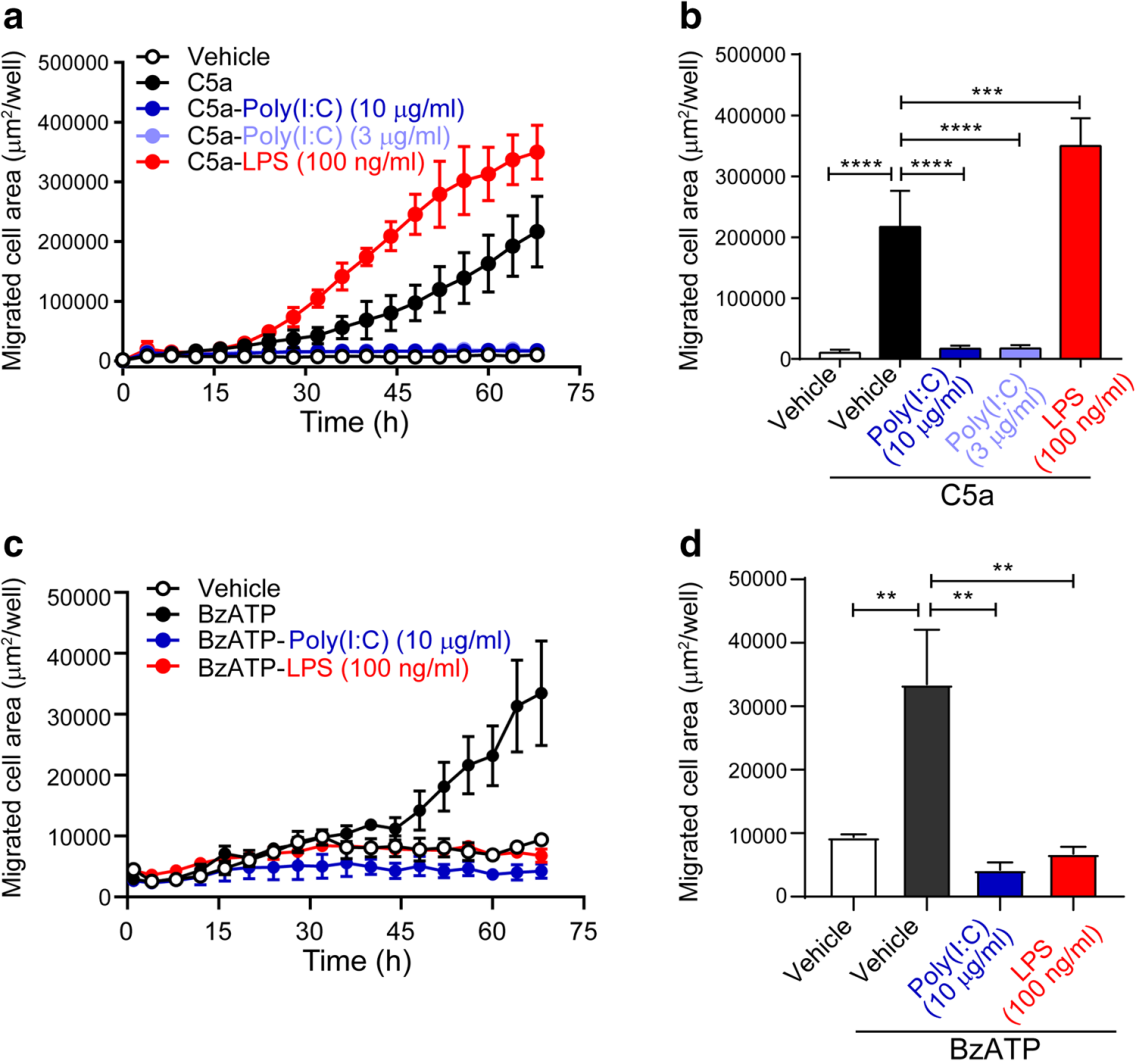
Chemotaxis of microglia in response to LPS or poly(I:C). Microglia treated with LPS or poly(I:C) were seeded into the upper chamber of plate inserts, while 4 nM C5a or 100 µM BzATP were added to the lower chamber. (**a**,**c**) Time-dependent chemotaxis of LPS- or poly(I:C)-treated microglia towards C5a (**a**) or BzATP (**c**) was monitored by Incucyte. (**b**, **d**) Area of migrated microglia towards C5a (**b**) or BzATP (**d**) was calculated at 68 h. Data are shown as means + SD. One-way ANOVA followed by the Tukey's post hoc test. **P < 0.01; ***P < 0.001; ****P < 0.0001.

Under pathological conditions, microglia activate and directionally migrate toward the site of injury guided by chemoattractants^32^. The complement component C5a has been proved as one of the chemoattractants to trigger direct microglia migration^29^. Our functional analysis based on differentially expressed genes revealed chemotactic differences between LPS- and poly(I:C)-treated microglia. To verify this hypothesis, we monitored chemotactic migration of non-treated or treated microglia in response to 4 nM C5a by Incucyte Zoom, a time-lapse live-cell image analysis system. Results showed that non-treated microglia migrated towards C5a in a time-dependent manner (Fig. 5a). Compared to non-treated controls, LPS enhanced chemotactic migration occurring from 28 h till 68 h, as indicated by the migrated cell areas (Fig. 5a). To our surprise, however, both 3 and 10 µg/ml poly(I:C) completely suppressed such function for the whole monitoring period (Fig. 5a). Since LPS-treated microglia achieved the greatest migration at 68 h, we further plotted the migrated areas at this time point. Again, the results demonstrated that C5a triggered microglia migration. In addition, 100 ng/ml LPS increased microglial chemotaxis towards C5a by 50%, while poly(I:C) completely inhibited C5a-induced microglial chemotaxis (Fig. 5b). To confirm the results, we tested microglial chemotaxis with another known chemoattractant, BzATP, which is a stable analog of ATP. Indeed, BzATP induced microglial migration starting at 36 h till 68 h within the monitoring time range (Fig. 5c). In contrast to the data from C5a-induced migration, both LPS- and poly(I:C)-treated cells exhibited reduced migration area towards BzATP (Fig. 5c). The reduction level was as similar as that under no-BzATP condition at 68 h (Fig. 5d). This indicates a full suppression by LPS and poly(I:C) in BzATP-induced chemotaxis. Therefore, we proved that LPS and poly(I:C) modulated chemotactic motility of microglia differently, which also depended on the chemoattractants used.

### Microglial phagocytosis in response to LPS and poly(I:C)

**Figure 6:**
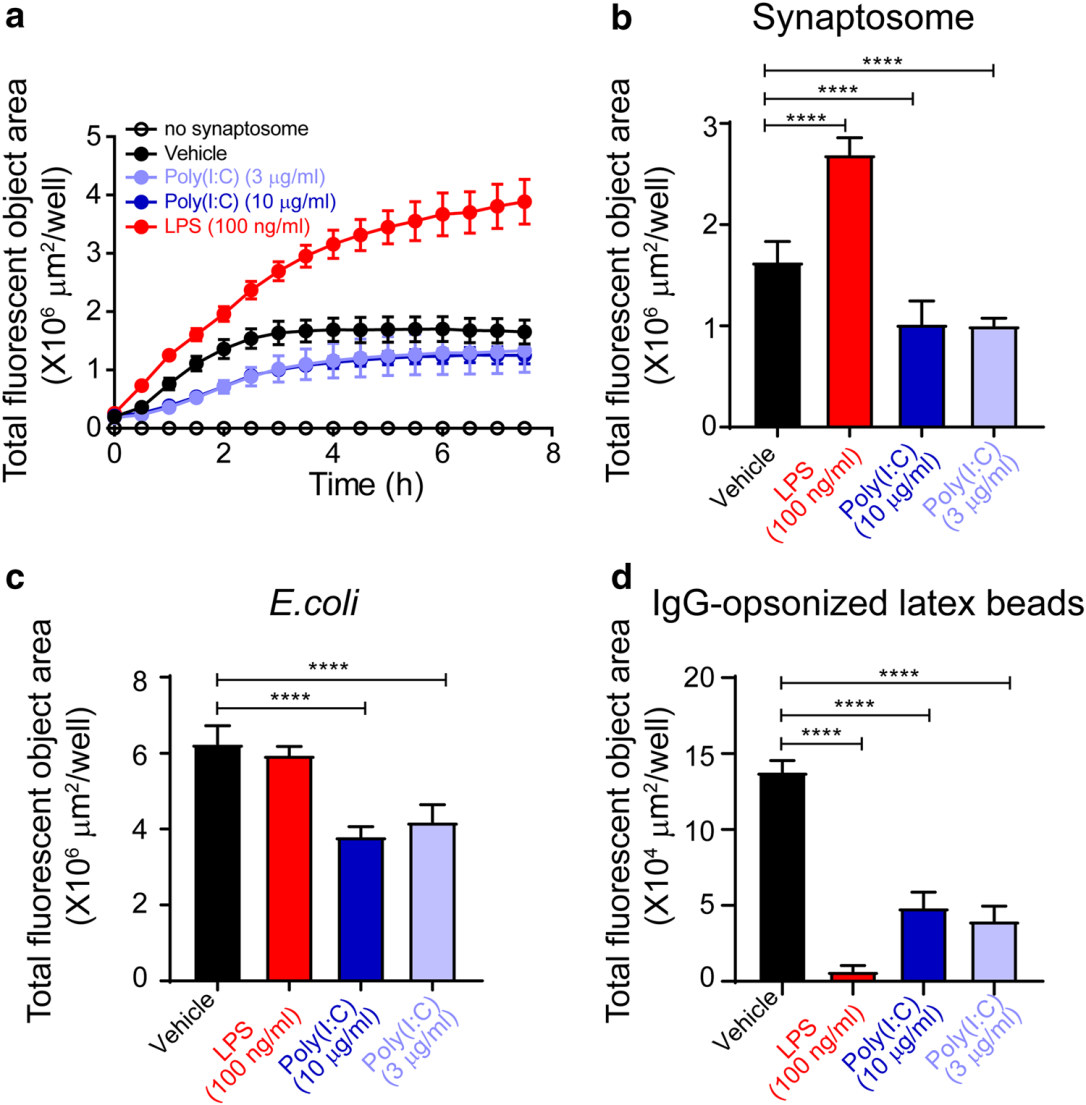
Phagocytosis of microglia in response to LPS or poly(I:C). Microglia were treated with either 100 ng/ml LPS, 10 µg/ml poly(I:C), or 3 µg/ml poly(I:C) for 24 h before being fed with different substrates. (**a**) Time-dependent uptake of pHrodo-labeled synaptosomes in LPS- or poly(I:C)-treated microglia was monitored by Incucyte. (**b**) Fluorescent area in microglia fed with pHrodo-labeled synaptosomes was quantified at 3 h. (**c**) Fluorescent area in microglia fed with pHrodo-labeled *E. coli* was quantified at 5 h. (**d**) Fluorescent area in microglia fed with pHrodo-labeled IgG-opsonized latex beads was quantified at 1 h. Data are shown as means + SD. Experiments were conducted in six replicates and repeated three times independently. One-way ANOVA followed by the Tukey's post hoc test. ****P < 0.0001.

Microglia prune synapses during development and diseases^33^. To examine how LPS and poly(I:C) modulate microglial pruning process, LPS- and poly(I:C)-treated microglia were fed with phrodo-labeled synaptosomes that were isolated from mouse brains. Then the engulfment was monitored with the Incucyte system. As expected, microglia phagocytosed synaptosomes time-dependently and reached the plateau at around 3 h, as indicated by fluorescent area (Fig. 6a). Compared with non-treated microglia, those treated with LPS showed an increase in fluorescent area, indicating an enhanced phagocytosis. Nevertheless, both 3 and 10 µg/ml poly(I:C) suppressed phagocytosis, evidenced by reduced fluorescent area within the treated cells (Fig. 6a). When quantifying the total fluorescent area at 3 h, we observed that LPS induced an about 50% increase in fluorescent area in microglia, but treatments with poly(I:C) at both 3 and 10 µg/ml resulted in a reduction by 30% (Fig. 6b). To further verify the effects of LPS and poly(I:C) on microglial phagocytosis, we fed LPS- and poly(I:C)-treated cells with various substrates, such as pHrodo-labeled *E. coli* and IgG-opsonized latex beads, and then measured their phagocytotic responses. Unlike the synaptosome data, we found that compared to non-treatment control, microglial phagocytosis of *E. coli* was not affected by LPS, but significantly suppressed by poly(I:C) at both concentrations (Fig. 6c). Interestingly, when fed with IgG-opsonized latex beads, both LPS- and poly(I:C)-treated microglia exhibited more than 60% reduction in phagocytosis, especially a 90% reduction in LPS-treated cells (Fig. 6d). Together, microglial phagocytosis was differentially affected by LPS and poly(I:C) and this differentiation also relied on the substrates fed to microglia, which further supported our gene-based analysis of microglial functions.

### Signaling pathways in microglia in response to LPS and poly(I:C)

**Figure 7:**
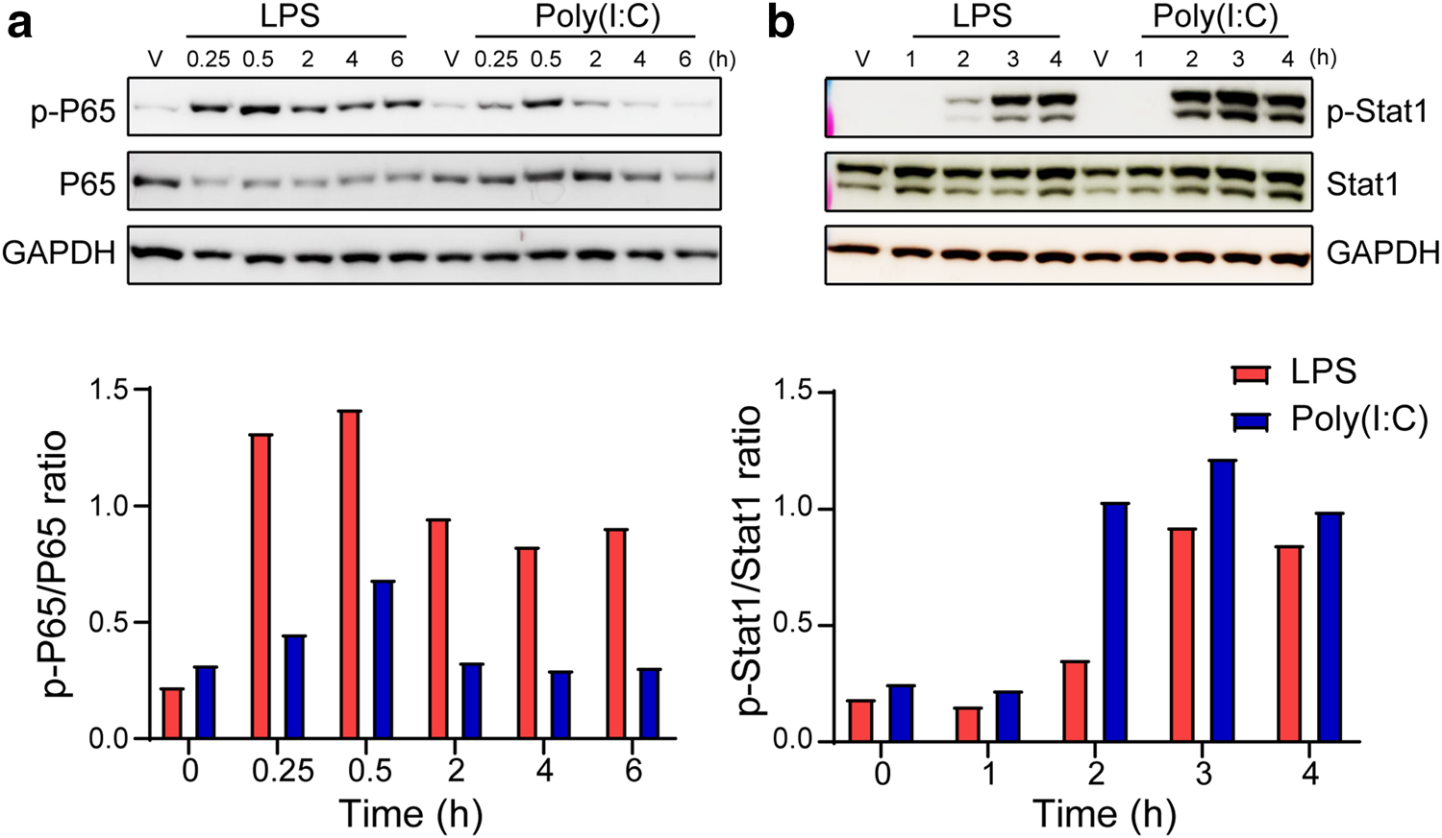
Downstream signaling in microglia induced by LPS or poly(I:C). Microglia were treated with 100 ng/ml LPS or 10 µg/ml poly(I:C) for different time intervals. (**a**) Western blots were sequentially probed with the antibodies against p-P65, P65, and GAPDH from the same gel. The histogram below represents quantification of p-P65 signal intensity that was normalized to P65. (**b**) Western blots were sequentially probed with the antibodies against p-Stat1, Stat1, and GAPDH from the same gel. The histogram below represents quantification of p-Stat1 signal intensity that was normalized to Stat1.

Although LPS and poly(I:C) led to different phenotypic changes in microglia ranging from morphology, proliferation, transcriptional regulation, secretion to chemotactic migration and phagocytosis, their downstream cascades are not clearly defined. NF-κB and JAK-STAT pathways are the major downstream signaling of TLRs^34^. Hence, we further compared the two pathways between LPS- and poly(I:C)-treated microglia by detecting phosphorylation of P65 for NF-κB pathway and phosphorylation of Stat1 for JAK-STAT pathway with Western blot. We performed a time-course study in microglia in the presence of 100 ng/ml LPS and 10 µg/ml poly(I:C). The results revealed a rapid phosphorylation of P65 within 15 min upon LPS stimulation, which reached the peak at 30 min and persisted for 6 h (Fig. 7a). In contrast, in poly(I:C)-treated microglia, the signal of phosphorylated P65 was not as strong as in LPS-treated cells and diminished at 6 h after reaching the peak at 30 min (Fig. 7a). Unlike phosphorylated P65, Stat1 was phosphorylated at 2 h upon stimulation of both LPS and poly(I:C), then reached the peak at 3 h and persisted for up to 4 h (Fig. 7b). The phosphorylation level of Stat1 was higher in poly(I:C)-treated microglia than in LPS-treated microglia in general, particularly at the 2-h time point (Fig. 7b). Full-length blots were also shown (Supplementary Fig. S3). Overall, the results indicated signaling differences in microglia in response to LPS and poly(I:C) stimulation, which may contribute to consequential differences in microglial functions.

### Co-culture response to LPS and poly(I:C)

**Figure 8:**
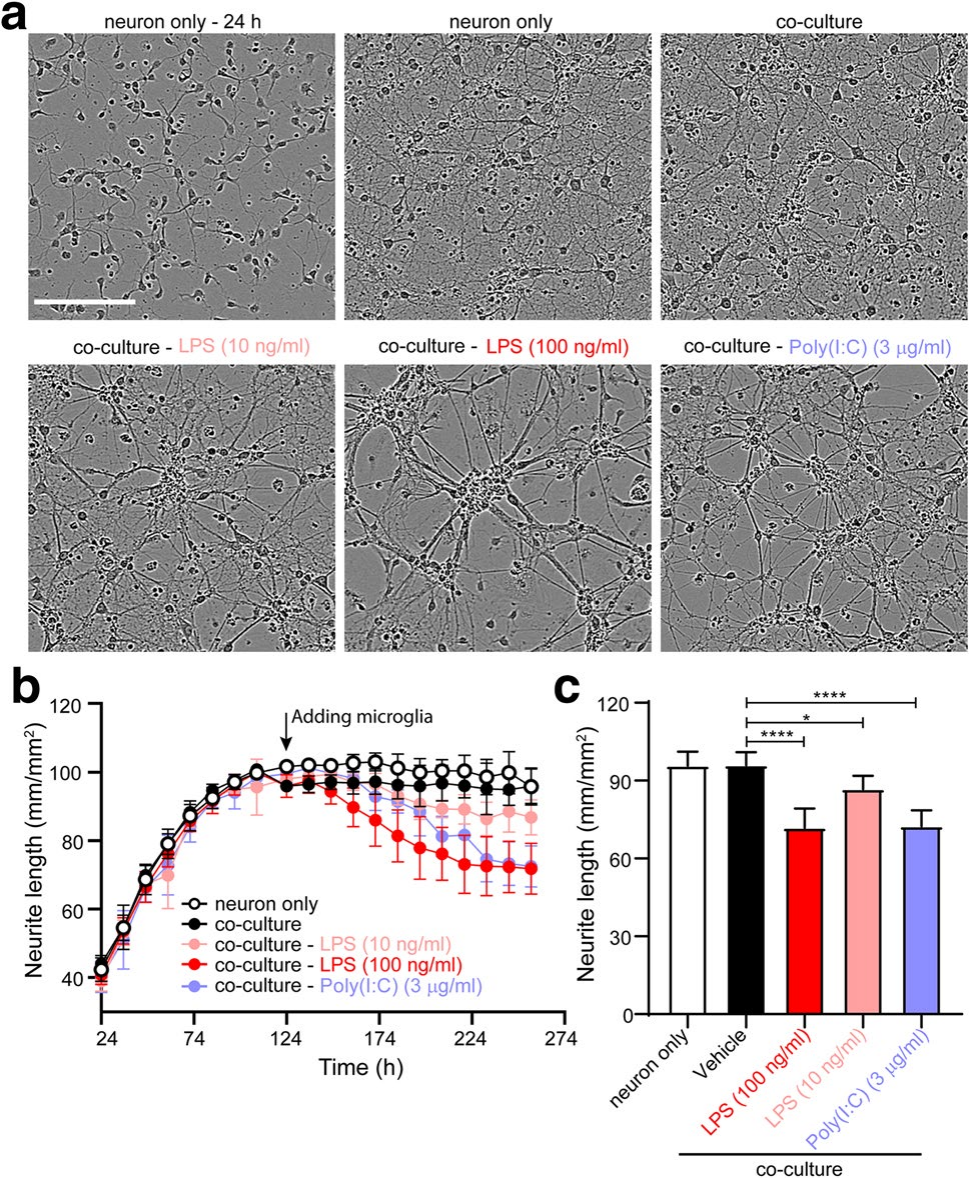
Neurite length affected by LPS- and poly(I:C)-treated microglia in a co-culture system. Neurons were cultured for around 6 days before microglial addition with or without LPS or poly(I:C) treatments for another 4 days. (**a**) Representative phase images of cells at the beginning time point (specified as “24 h”) and last time point (256 h) in the monitoring time range under the indicated conditions. Scale bar, 150 µm. (**b**) Time-dependent neurite outgrowth in LPS- or poly(I:C)-treated co-culture system was monitored by Incucyte. Arrow indicates the time of adding microglia. (**c**) Neurite length under the indicated conditions was measured at 256 h. Data are shown as means + SD. Experiments were conducted in six replicates and repeated two times independently. One-way ANOVA followed by the Dunnett's post hoc test. *P < 0.05; ****P < 0.0001.

We have demonstrated distinct biological functions and signaling pathways in LPS- and poly(I:C)-treated microglia. Next, we would like to explore how these treated microglia interact with neurons. To that end, we isolated and cultured neurons for around 6 days, followed by adding treated or non-treated microglia to establish a co-culture system, in which the number of microglia was 20% of the number of neurons to best mimic cell type composition in the brain. During the culture, neurons outgrew their processes and generated neuronal networking under the “neuron only” and non-treated co-culturing conditions (Fig. 8a). However, after addition of LPS- or poly(I:C)-treated microglia into the co-culture system, neurons displayed diminished neurites, destroyed networking, and clustered cell bodies (Fig. 8a), indicating neurotoxicity mediated by LPS- or poly(I:C)-activated microglia. We further measured neurite length to characterize the level of neurotoxicity. Consistent with the microscopic observations, the neurites continued to grow until the time of adding microglia, while the co-culture with 100 ng/ml LPS- or 3 µg/ml poly(I:C)-activated microglia caused a reduction of more than 25% in neurite length, as compared to non-treated controls (Fig. 8b,c). These findings were neither observed under the “neuron only” condition, nor under the non-treated co-culturing condition, where the neurite length kept plateau after microglial addition (Fig. 8b). In summary, both LPS- and poly(I:C)-activated microglia caused neurotoxicity to co-cultured neurons, suggested by aberrant neuronal morphology and decreased neurite length, which may be mediated by separate underlying mechanisms.

## Discussion

Microglia become activated through recognizing PAMPs including bacterial endotoxin LPS and viral dsRNA poly(I:C), and the persistent activation leads to a variety of inflammatory phenotypes. In this study, we compared the phenotypes in microglia in response to LPS and poly(I:C), ranging from morphology and proliferation to chemotaxis and phagocytosis. Furthermore, we investigated transcriptome and downstream signaling cascades in microglia upon LPS and poly(I:C) stimulation; the results suggested a cellular mechanism for the phenotypic changes. Last, we explored neurotoxicity mediated by LPS- and poly(I:C)-treated microglia in a co-culture system.

Morphological changes in microglia are used to discriminate among different activation states both in vivo and in vitro^35^. In the resting or inactive state, microglia present a ramified morphology with long and fine processes, while stimulation with classical activation factors such as LPS results in retraction of microglial processes and development of an amoeboid phenotype. Indeed, in our study, LPS transformed microglial morphology from a polarized baseline state to an ameboid-like state. However, to our surprise, microglia stimulated by poly(I:C) exhibited a bushy morphology with shorter and thicker processes distributed around swelling cell bodies. This bushy morphology was also observed in rat microglia after infusing the brain with poly(I:C)^36^, as well as in microglia cultured on organotypic hippocampal slice following poly(I:C) stimulation^37^. So far, however, it is not clear if the bushy morphology is an intermediate form between the polarized and ameboid-like forms, or an independent state separated from the other two states, and how these distinct morphologies and activation states link to microglial functions. Further investigation may reveal insights regarding these issues.

In parallel to morphological assessments, we compared migration and phagocytosis of microglia in response to LPS and poly(I:C). Interestingly, we found that in contrast to LPS that facilitated microglial migration towards C5a, poly(I:C) suppressed these functions. On the other hand, both LPS and poly(I:C) fully blocked microglial migration towards BzATP. In previous studies, inconsistent results were also observed in LPS-induced migration. It has been reported that LPS increased migration towards FBS in RAW264.7, a macrophage cell line^38^, and in primary rat macrophages^39^; while some studies showed that there were no changes in migration towards serum in BV2 microglial cell line by LPS^40^, and even that LPS could impair migration towards ATP in primary rat^41^ and human microglia^42^, which was consistent with our BzATP data. These disagreements of the effects of LPS on microglial migration may depend on species, cell types, and categories and concentrations of chemoattractants, which was supported by the observed distinct effects of LPS on C5a- and BzATP-induced cell migration.

There are various receptors and pathways involved in microglial phagocytosis including those participating in Fc receptor- and complement-mediated phagocytosis. It has been demonstrated that not only LPS but also poly(I:C) enhanced microglial phagocytosis of fungi, which may be dependent of MyD88 or TRIF^43^. Poly(I:C) has also shown to increase TRIF-mediated clearance of axonal debris in microglia^44^. In our study, LPS-treated microglia showed enhancement, no change, and inhibition in engulfing synaptosome, *E. coli,* and IgG-opsonized latex beads, respectively; while poly(I:C) suppressed all these phagocytosis processes. These conflicting phagocytotic responses of microglia across our data and previous reports may rely on the substrates used, which represent their independent phagocytotic pathways mediated by separate receptors.

The differences between inflammatory responses to LPS and poly(I:C) are attributed to the activation of microglia through distinct TLRs. LPS is recognized through TLR4, while poly(I:C) is recognized through TLR3. Consistent with other reports investigating BV2 cells and macrophages^6,27,28^, we found that binding to different TLRs can initiate distinct gene expression, protein release, and signal pathways in primary microglia as well. For example, in our studies, LPS induced high expression of IL6 and IL1β both at mRNA and protein levels in microglia, which was the consequence of NF-κb pathway activation. Meanwhile, a considerable type-I IFN response at both mRNA and protein levels was observed in microglia when treated with poly(I:C), subsequently activating p-Stat1 signaling. Our findings agree with the previous report that NF-κb and STAT1 pathways were significantly enriched in LPS- and poly(I:C)-activated BV2 cells^6^. These data not only reveal different underlying mechanisms upon stimulation by LPS and poly(I:C), but also elucidate the Western blot results that NF-κB pathway was activated at early time points whereas STAT1 pathway was detected at late time points. Our further biological function analysis based on the differentially expressed genes upon LPS and poly(I:C) induction suggested there exist differences in a few microglial functions, such as proliferation, chemotaxis, and phagocytosis. This analysis not only mirrored our experimental results, but provided genetic evidence explaining these differences. However, to dissect the roles of TLRs, NF-κb and STAT1 pathways in microglial phenotypes, knockout of TLRs or blockade of either pathways is needed for future studies.

To better understand cellular crosstalk and mimic in vivo neuroinflammation, we developed a co-culture system consisting of microglia and neurons, which allows physical cell–cell interaction and communication through secreted proteins. To our interest, although LPS and poly(I:C) showed distinct effects on microglial TLR recognition, biological functions, and downstream signals, the microglia activated by these two stimuli caused neurotoxicity to neighboring neurons in the co-culture system. As TLR3 and TLR4 are not present in neurons^45^, the neurotoxicity is most likely due to the activated microglia. The results are consistent with those in the in vivo neuroinflammation models induced by LPS or poly(I:C), in which presynaptic disruption and neurodegeneration was induced and mediated by activated microglia^46,47^. Nevertheless, it is unknown whether the effects in our model were triggered by direct interaction with microglia or by indirect communication through microglia-secreted proteins, which still needs to be further explored.

Although lots of work has reported the individual effects of LPS or poly(I:C) on microglial functions^22–26^, our study systemically compared inflammatory responses in primary microglia upon LPS and poly(I:C), and demonstrate the similarity and uniqueness in their downstream genes and pathways. Our results are complementary to previous studies and may broaden our knowledge of neuroinflammation. Moreover, the current findings regarding transcriptional changes, secretion, signaling pathways, and neurotoxicity are well in line with previous reports of in vivo neuroinflammation models induced by LPS and poly(I:C)^24,26,46,47^, which suggests our in vitro tools are useful for modeling the behavior of in vivo cells and dissecting individual cellular responses. These in vitro results would therefore assist in prediction and explanation of in vivo microglial phenotypes induced by LPS and poly(I:C). Furthermore, it provides a basis for determining suitable in vitro neuroinflammation models, which can be potentially and effectively utilized for drug discovery. There are two more directions for future investigation. First, the primary microglia in our study are of postnatal origin to ensure high yield for requirements of functional and mechanism studies. Since microglial transcriptome and phenotypes vary with development and aging^48,49^, comparative studies with microglia from adult tissues would be worthy to implement. Second, this study is performed by using primary cultured cells. However, in vitro culture conditions may not exactly reflect physiological environment in the brain, possibly impacting gene expression profile in cultured microglia compared to their naïve counterparts^50^. Although we developed a co-culture system with microglia and neuron, which better mimics the in vivo situation than mono-culture system and may untangle some complex questions of cell–cell interaction, we cannot exclude the contribution of other essential cell types in the brain such as astrocytes and endothelial cells. So further validation with in vivo studies are critical for development of microglia-targeting therapeutics.

## Methods

### Animals

Wild-type C57BL/6 time-pregnant mice were obtained from Charles River laboratories, Inc. Mice were allowed to acclimate for 7 days after receipt. They were kept on a 12-h light/dark cycle and allowed free access to food and water. All animal care and use complied with the Guide for the Care and Use of Laboratory and all experimental protocols were approved by the IACUC. The study was carried out in compliance with the ARRIVE guidelines (https://arriveguidelines.org).

### Reagents

LPS (O55:B5) and Poly(I:C) (HMW) were purchased from Sigma-Aldrich and Invivogen, respectively.

### Mouse microglia isolation and culture

Cortices from P0-2 C57BL/6 mouse pups were dissected, stripped of meninges, mechanically dissociated with a hand homogenizer and a 25-gauge needle. The cell suspension was seeded into poly-l-lysine-coated (Sigma-Aldrich) T150 tissue culture flasks and maintained in DMEM/F12 with 10% FBS and 1% penicillin–streptomycin for 10–14 days to grow a confluent mixed astrocyte/microglia population. We trypsinized, gently scraped, and collected the cells. Then we applied the cells to an antigen–antibody–mediated magnetic cell-sorting (MACS, Miltenyi Biotech) assay to positively select microglia as previously described^51^. Briefly, the mixed glial population was re-suspended in MACS buffer (Miltenyi Biotech) and incubated with CD11b MicroBeads (Miltenyi Biotech). The cell suspension was then applied to LS separation column (Miltenyi Biotech) fitted into a QuadroMACS cell separator (Miltenyi Biotech). Unlabeled cells could pass through the column while labeled cells remained captured in the magnetic field. After washing the column with MACS buffer, the column was then removed from the magnetic separator and flushed with MACS buffer to collect the purified microglia population. For an increased level of purity, the eluted microglia population was passed through a new LS separation column a second time. The purity of microglia used in our study was more than 95% assessed by immunocytochemistry (data not shown).

### Immunocytochemistry

Method was described before^29^. Briefly, fixed and permeabilized cells were blocked with 10% donkey serum, then were probed with primary antibodies (Iba1, 1:1000, WAKO Chemicals; GFAP, 1:1000, Abcam) for 2 h followed by incubation with fluorochrome-conjugated secondary antibodies (Alexa Fluor 488 and 555, 1:200, Molecular Probes, respectively). Nuclei were counterstained with DAPI. A confocal-laser microscope (LSM 700; Carl Zeiss MicroImaging) was used to acquire images with a multi-track configuration.

### Microglial morphology analysis and proliferation

As previously described^29,35^, we defined amoeboid-like microglia as flat Iba1^+^ cells without thin processes while polarized microglia with thin processes, and bushy microglia with short but sturdy, multiple processes. We then manually counted the number of cells with various morphologies and calculated the percentages of amoeboid-like, bushy, and polarized microglia in total microglial cells under different conditions. At least 5 randomly selected fields were used for quantification.

Cell number was determined by cell counting kit-8 (CCK-8, Dojindo), which measures mitochondrial dehydrogenase activity inside the cells. Briefly, 10 μl of CCK-8 solution was added to 100 μl of media in each well of the plate. After incubating the plate for 2–4 h at 37 °C, the absorbance at 450 nm was measured using the Bio-Rad microplate reader.

### RNA extraction and quantitative real-time PCR

Microglia were homogenized and total RNA was extracted using RNeasy plus mini kit (Qiagen). Total RNA concentrations were measured using NanoDrop ND-1000 spectrophotometer. RNA was reverse transcribed into cDNA using Superscript III reverse transcriptase (Invitrogen) with random hexamer primers. Transcript abundance was determined by using SYBR Green PCR mix (Applied Biosystems), with primer pairs against different genes. The following primer pairs were used for qRT-PCR:

- *Gapdh*: 5′ AGGTCGGTGTGAACGGATTTG 3′ (F) and 5′ TGTAGACCATGTAGTTGAGGTCA 3′ (R)
- *Ptgs2*: 5′ TTCAACACACTCTATCACTGGC 3′ (F) and 5′ AGAAGCGTTTGCGGTACTCAT 3′ (R)
- *Il6*: 5′ TAGTCCTTCCTACCCCAATTTCC 3′ (F) and 5′ TTGGTCCTTAGCCACTCCTTC 3′ (R)
- *Tnfa*: 5′ CCCTCACACTCAGATCATCTTCT 3′ (F) and 5′ GCTACGACGTGGGCTACAG 3′ (R)
- *Tspo*: 5′ GCCTACTTTGTACGTGGCGAG 3′ (F) and 5′ CCTCCCAGCTCTTTCCAGAC 3′ (R)
- *Nos2*: 5′ GTTCTCAGCCCAACAATACAAGA 3′ (F) and 5′ GTGGACGGGTCGATGTCAC 3′ (R)
- *Ifna*: 5′ GGACTTTGGATTCCCGCAGGAGAAG 3′ (F) and 5′ GCTGCATCAGACAGCCTTGCAGGTC 3′ (R)
- *Ifnb*: 5′ CAGCTCCAAGAAAGGACGAAC 3′ (F) and 5′ GGCAGTGTAACTCTTCTGCAT 3′ (R)

### RNA sequencing and data processing

RNA quality was assessed by using Agilent RNA 6000 Nano Kit and Agilent 2100 Bioanalyzer before sequencing by BGI, a fee-for-service provider. Samples with RNA integrity number (RIN) above 9 were sequenced. Data processing was performed on QIAGEN OmicSoft Suite (version 10.0, https://digitalinsights.qiagen.com/products-overview/discovery-insights-portfolio/qiagen-omicsoft/), as described previously^29^. Reads were mapped to mouse GRCm38 genomes (https://www.ncbi.nlm.nih.gov/grc/) using OmicSoft sequence aligner (OSA) of the OmicSoft Suite software. Gene expression read count and TPM (Transcript Per kilobase Million) were calculated based on mouse RefSeqGene gene model (https://www.ncbi.nlm.nih.gov/refseq/rsg/). Samples in each group were QCed based on overall gene expression consistency, and outliers were removed before downstream analysis.

We deposited the RNA-sequencing data in NCBI with BioProject ID PRJNA615297.

### Differential gene expression and pathway analysis

Read depth differences between samples and gene expression differences among groups were analyzed by inference tests based on the Voom algorithm. Before the inference tests, genes with low or no expression (average TPM < 0.1) were excluded. Genes with expression changes of more than fourfolds and adjusted *P* value (calculated by Benjamin–Hochberg procedure) of less than 0.05 from the inference test were selected as differentially expressed genes.

GeneOntology analysis (http://geneontology.org/) was applied to analyze the enrichment of differentially expressed genes in biological pathways and processes. Enrichment of significant pathways (adjusted *P* value < 0.05, calculated by the database) in each analysis were exported from the database and charted using ArrayStudio version 10.0 or Excel.

### Secretome analysis

Microglia were treated with either LPS (10 ng/ml and 100 ng/ml) or poly(I:C) (3 µg/ml and 10 µg/ml) for 24 h. Then, the relative concentrations of secreted proteins in cell supernatants were measured by using antibody-based 29-plex immunoassays (Luminex, R&D systems) as described before^29^. The 29 measured proteins were: CCL2/JE/MCP1, CCL3/MIP1α, CCL4/MIP1β, CCL5/RANTES, CCL20/MIP3α, CCL22/MDC, CXCL1/KC, CXCL2/MIP2, CXCL10/IP10/CRG2, FGFβ, GCSF, GMCSF, IGFI, IL1α, IL1β, IL4, IL6, IL9, IL10, IL12 p70, IL13, IL17A, IL23 p19, IL33, MCSF, RAGE, Resistin, TNFα, VEGF.

### Chemotaxis

Inserts of the culture plates (Sartorius) were precoated with ICAM. LPS- or poly(I:C)-treated cells in DMEM/F12 containing 0.5% FBS were seeded into the upper chamber. The lower chamber was filled with 4 nM C5a or 100 µM BzATP. IncuCyte Zoom live-cell system (Sartorius) was used to monitor chemotaxis every four hours for 68 h.

### Synaptosome preparation, IgG-opsonized latex bead preparation, and phagocytosis

Mice were perfused transcardially with phosphate buffer before brain dissection. Then whole brain was homogenized in Syn-PER Reagent (ThermoFisher Scientific) and synaptosomes were pelleted after centrifuge at 15,000*g*. Purified synaptosomes were quantified and then labeled with pHrodo Red dye by using pHrodo iFL Red Microscale Protein Labeling Kit (ThermoFisher Scientific).

Aliphatic amine latex beads (3 µm, Thermofisher) were incubated with 3 mg/ml mouse IgG (Thermofisher) on a rotator overnight at 4 °C to allow proper binding. After washing to remove any unbound IgG, IgG-opsonized latex beads were labeled with pHrodoRed, succinimidyl ester (Thermofisher) for 1 h at room temperature, followed by repeated wash to remove free dye.

Microglia treated with LPS or poly(I:C) for 24 h were fed with pHrodo-labled synaptosomes, pHrodo-labeled *E. coli* (Sartorius), or pHrodo-labeled IgG-opsonized latex beads. Fluorescence was monitored every half an hour for 7.5 h by IncuCyte Zoom live-cell system (Sartorius).

### Western blot

RIPA buffer (Amresco) with protease and phosphatase inhibitors (Sigma and Roche respectively) were applied to homogenize and lyse treated cells. The immunoblot method was described previously^51^. The antibodies used were p-P65 (1:1000, Cell Signaling Technology), p-Stat1 (1:1000, Cell Signaling Technology), P65 (1:000, Cell Signaling Technology), Stat1 (1:1000, Cell Signaling Technology), and GAPDH (1:1000, Millipore).

### Co-culture system and neurite monitoring

Primary mouse neurons were isolated from the cortices of day 18 embryonic mouse brains. After meninge removal, the brain tissues were digested into single cells by using Neural Tissue Dissociation Kit (Miltenyi) according to the instruction, followed by Neuron Isolation Kit (Miltenyi) to obtain relatively pure neurons. The isolated neurons were cultured for 6 days in NbActiv4 media (BrainBits) before the addition of primary microglia with or without LPS or poly(I:C) treatments. The number of microglia was 20% of the number of neurons. Cell morphology was monitored the next day of culture and scanned every 4 h by IncuCyte Zoom live-cell system (Sartorius) and neurite length was analyzed by IncuCyte NeuroTrack software module (Sartorius).

### Statistical analysis

Data analysis was performed by using one-way ANOVA and Tukey's or Dunnett's post hoc test among groups with GraphPad Prism 8 (GraphPad Software, Inc.). *P* < 0.05 was considered statistically significant.

## Supplementary Information

Supplementary Information 1.

Supplementary Information 2.
