# Supplementary material for: Mouse primary microglia respond differently to LPS and poly(I:C) in vitro
Source: Sci Rep. 2021 May 17;11:10447. doi: 10.1038/s41598-021-89777-1 (PMC8129154; doi:10.1038/s41598-021-89777-1)
Supplement: Supplementary file 1 — Supplementary Information 1. [file 41598_2021_89777_MOESM1_ESM.docx]

**Supplementary Information**

Mouse primary microglia respond differently to LPS and poly(I:C) *in vitro*

Yingbo He^1,^*, Natalie Taylor^1^, Xiang Yao^2^, Anindya Bhattacharya^1^

^1^ Janssen Research & Development, LLC., Neuroimmunology Drug Discovery, San Diego, California

^2^ Janssen Research & Development, LLC., NonClinical Safety, San Diego, California

*Address correspondence to Yingbo He, Janssen Research & Development, LLC., Neuroimmunology Drug Discovery, 3210 Merryfield Row, San Diego, CA 92121. E-mail: [yhe50@its.jnj.com](mailto:yhe50@its.jnj.com)


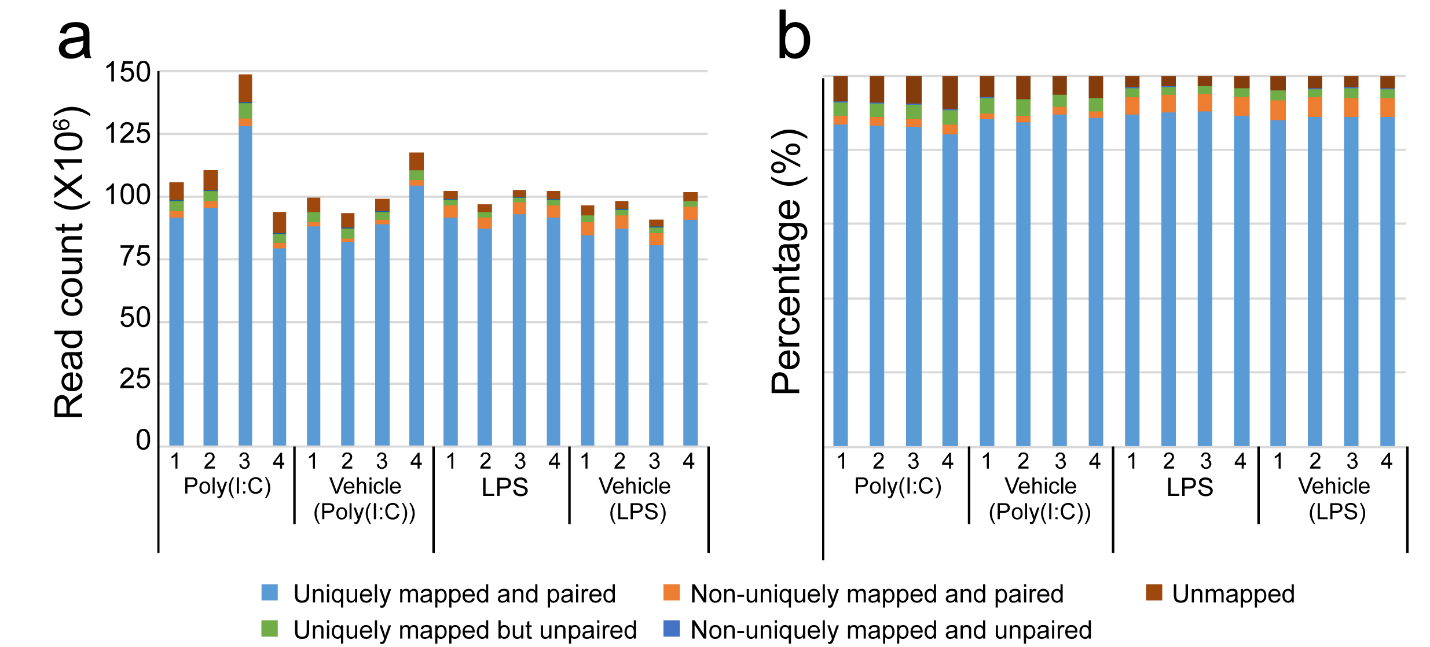


**Supplementary Fig. S1: Sequencing depth of all the RNA-seq samples. a** Read count of all samples. **b** Mapping percentage of all samples. Vehicle (LPS) indicates vehicle corresponding to LPS treatment group. Vehicle (poly(I:C)) indicates vehicle corresponding to poly(I:C) treatment group.


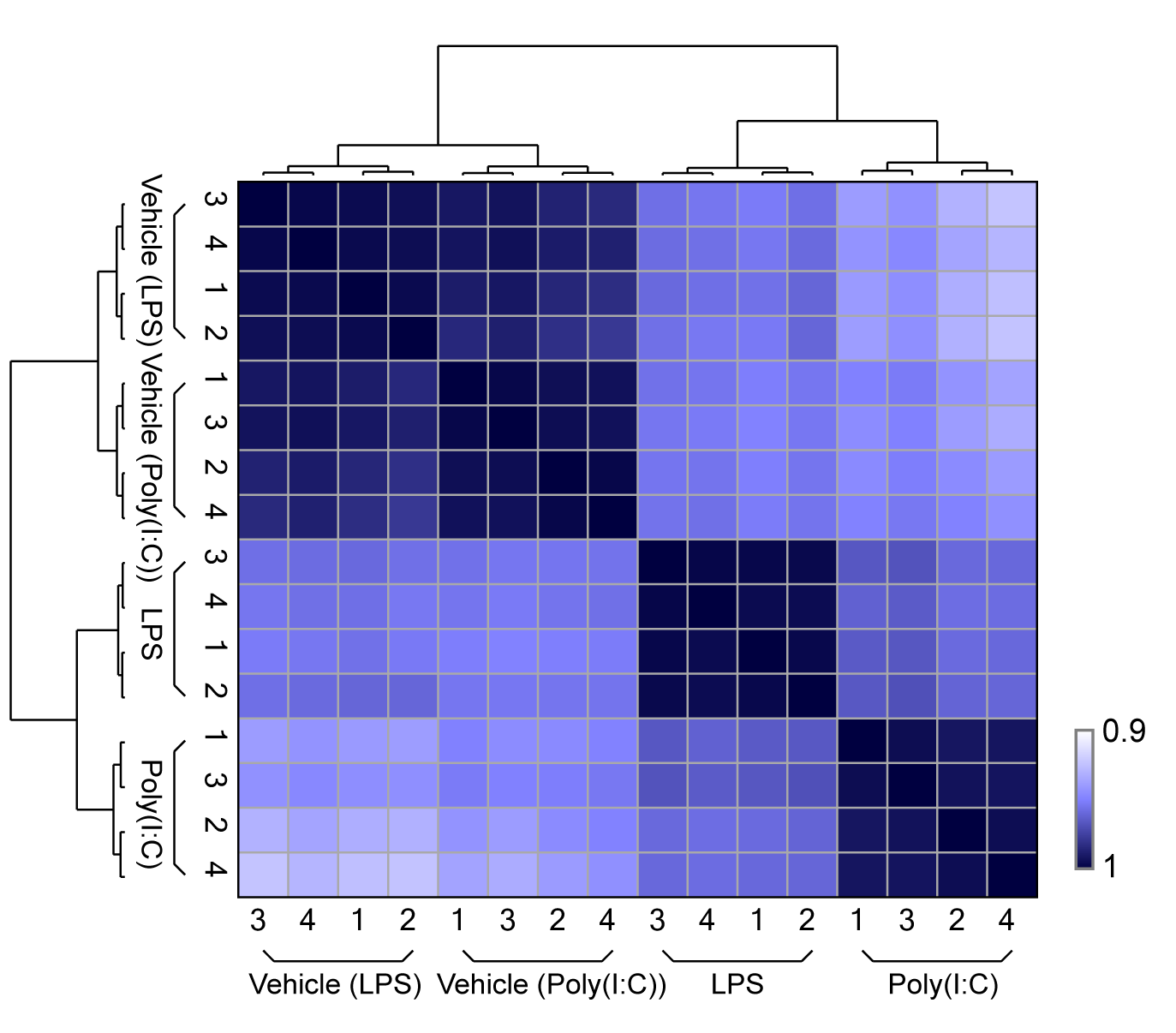


**Supplementary Fig. S2: Transcriptome analysis of all RNA-seq samples.** Heat map of pair-wise Pearson correlation between different treatment groups and biological independent replicates. Vehicle (LPS) indicates vehicle corresponding to LPS treatment group. Vehicle (poly(I:C)) indicates vehicle corresponding to poly(I:C) treatment group.

**
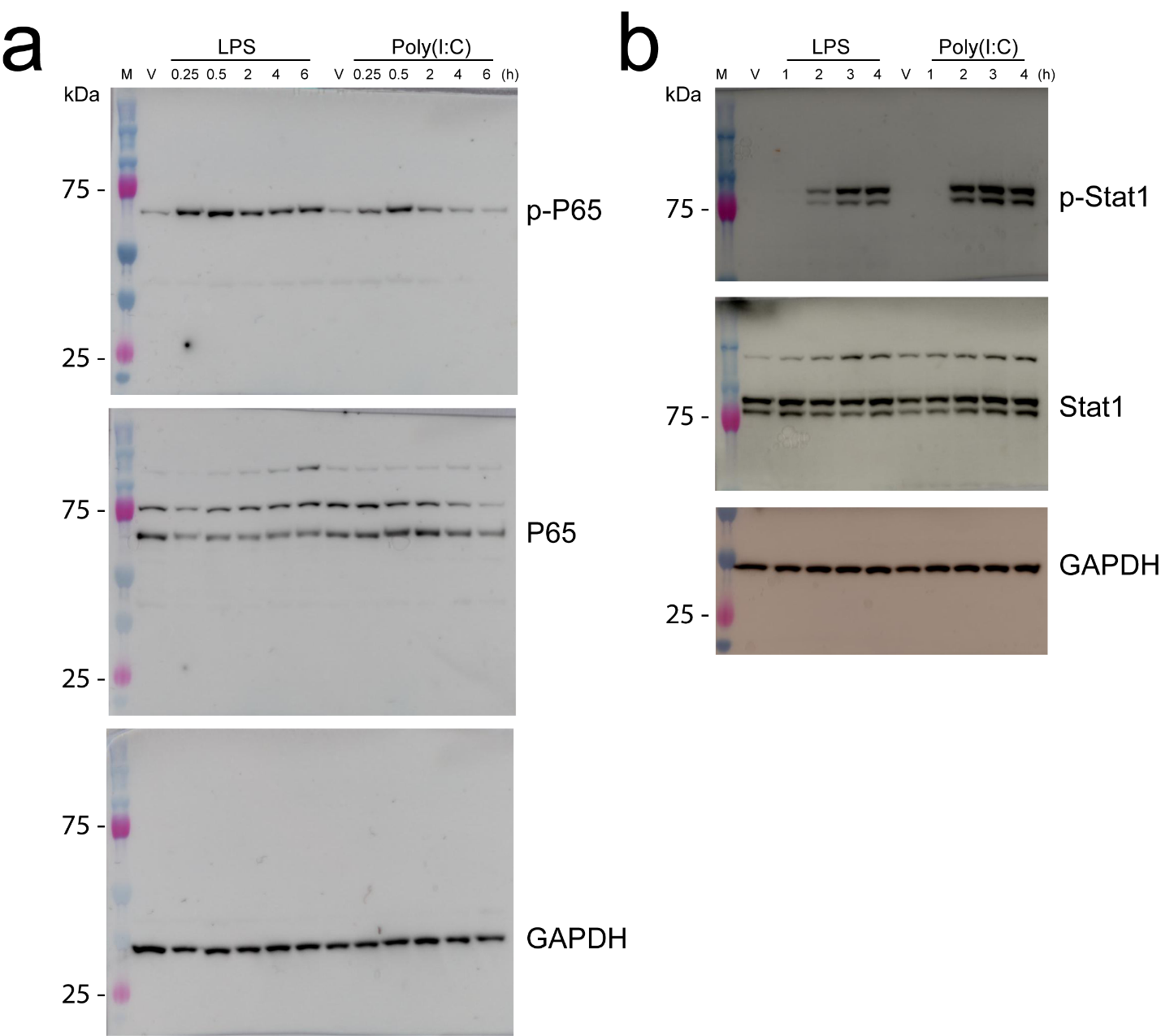
**

**Supplementary Fig. S3: Full-length blots presented in figures. a** Full-length blots of Fig.7a. All blots were from the same gel. **b** Full-length blots of Fig.7b. All blots were from the same gel but cut according to the molecular weight.

**Supplementary Table S1: List of top genes regulated by LPS and poly(I:C) in microglia.** LPS down-polyIC down: genes downregulated by both LPS and poly(I:C) in mciroglia; LPS up-polyIC up: genes upregulated by both LPS and poly(I:C) in mciroglia; LPS up-polyIC down: genes upregulated by LPS while downregulated by poly(I:C) in microglia; LPS down-polyIC up: genes downregulated by LPS while upregulated by poly(I:C) in microglia.
